# Unveiling a Bulk WTaV Multicomponent Alloy With Superior Thermal Properties and Manufacturability

**DOI:** 10.1002/advs.202522334

**Published:** 2026-04-01

**Authors:** Ishtiaque K. Robin, Skye N. Supakul, Shalini Tripathi, Eda Aydogan, Matthew T. Vigil, Caleb Hatler, Bochuan Sun, Akshay Korpe, Enrique Martinez, Saryu J. Fensin, Dan J. Thoma, Osman El‐Atwani

**Affiliations:** ^1^ Energy and Environmental Directorate Pacific Northwest National Laboratory Richland WA USA; ^2^ Materials Science and Engineering University of Wisconsin Madison Madison WI USA; ^3^ Materials Science and Engineering Clemson University Clemson SC USA; ^4^ School of Mechanical and Automotive Engineering Clemson University Clemson SC USA; ^5^ Los Alamos National Laboratory Los Alamos NM USA

**Keywords:** arc‐casting, characterization, high‐temperature, multi‐component alloys, STEM‐EDS, thermal conductivity

## Abstract

Many tungsten (W)‐based medium and high entropy alloys (HEA) demonstrate superior microstructural stability and enhanced mechanical properties as compared to pure W, effectively rendering them as viable candidate materials for extreme environments such as nuclear fusion, aerospace applications, and so on. However, service applicability of these alloys has two major challenges: (i) bulk manufacturing while maintaining desired phases, and (ii) inferior thermal performance. Here, we present a successfully manufactured arc‐cast WTaV multicomponent alloy based on input from thermodynamic and atomistic Monte‐Carlo simulations. A single‐phase body‐centered cubic (BCC) structure devoid of brittle intermetallics was produced with a microhardness value of ∼535 HV. A minimal reduction of microhardness (∼30 HV) after a 24 h‐1800°C heat‐treatment indicates not only high temperature thermal stability, but also enhanced mechanical properties. Remarkably, this alloy exhibits exceptional thermal conductivity (∼57 Wm^−^^1^ K^−^^1^ at room temperature and ∼134 Wm^−^^1^K^−^^1^ at 1000°C) surpassing that of W at high temperature, positioning it as one of the highest known multicomponent refractory alloys reported to date. Unlike W, which shows decreasing thermal conductivity with increasing temperature, this WTaV multicomponent alloy exhibits an opposite trend of increased thermal conductivity with temperature. The results clearly demonstrate potential candidacy of this material for demanding applications, providing an optimum balance of cost‐effectiveness, chemical simplicity, easier manufacturability, and acceptable thermal properties in the bulk form.

## 1 Introduction

Nuclear energy, particularly fusion, is a potential sustainable energy form to meet ever‐increasing energy demands with a minimal carbon footprint [1, 2]. However, the extreme environment present in fusion reactors requires structural materials that possess not only enhanced strength, thermal stability, and thermal conductivity at high temperature, but also show superior resistance to radiation damage [3, 4, 5]. Steels and nickel‐based superalloys are conventional structural materials known for reliable performance in many current industrial settings. Nonetheless, these materials face challenges under intense neutron irradiation and the extreme temperatures expected in fusion reactors [4, 5, 6, 7, 8]. Over the past decade, multicomponent alloys including high entropy alloys (HEAs) and medium entropy alloys (MEAs) have emerged as promising alternatives, offering enhanced properties due to their compositional complexity. The performance of these multicomponent alloys has been attributed to four core effects: (i) severe lattice distortion, (ii) high entropy effect, (iii) sluggish diffusion, and (iv) the cocktail effect resulting from the complex interactions between constituent elements [9, 10, 11, 12, 13, 14].

Multicomponent alloys, particularly those including refractory elements also known as refractory high entropy alloys (RHEA), are considered candidates for applications in high temperatures and radiation damage regimes [11]. Within refractory elements based multicomponent alloys, the presence of a complex energetic landscape originating from local lattice distortions and short‐range diffusion enhance microstructural and mechanical properties, including superior radiation tolerance, phase stability, and resistance to deformation, making them strong contenders for applications in harsh environments [14, 15]. However, manufacturing of these materials is costly, and it is not always possible to avoid formation of embrittling phases due to interaction between the elements present in the composition [15, 16]. MEAs are positioned between conventional alloys and HEAs with a suitable balance in cost, stability, strength, and design flexibility [12]. Achieving a single‐phase random solution in these alloys while maintaining enhanced mechanical properties along with a stable microstructure is comparatively easier due to fewer compatible elements [17, 18, 19]. Multi‐component alloys and MEAs with three or four elements reduce the possibility of formation of brittle intermetallic phases and compositional segregation during solidification, promoting stable solid‐solution microstructures [20, 21], which is often observed in high entropy alloys [22, 23]. Additionally, fewer elements in medium entropy based multi‐component alloys simplify the synthesis process and reduce challenges typically associated with excessive alloying, resulting in better cost‐effectiveness and desired mechanical properties [17, 24].

Manufacturing of multicomponent alloys through conventional means, particularly those containing W, remains a challenge due to a high amount of energy requirements and precise control of containment and temperature [25]. Bulk manufacturing has issues related to phase stability, microstructure inhomogeneity, and defect formation [26]. Heterogeneous microstructures, including microsegregation and non‐uniform grains, are often observed in arc‐cast RHEAs which is reported to be due to thermal gradients during solidification [27]. Additionally, different melting temperature of constituent elements also contributes to the inhomogeneity in these alloys [27, 28]. Here, to achieve desired properties of W‐based RHEAs while maintaining relatively simple chemistry, we considered a WTaV multi‐component alloy for synthesis through arc‐casting, followed by a homogenization heat treatment at 1800°C for 24 h. The micro‐hardness in the as‐cast state, coupled with negligible hardness reduction after heat‐treatment, highlights the successful synthesis and superior thermal stability of this alloy. The alloy design for the exact composition is established through CALPHAD [29] and atomistic Monte‐Carlo simulations. Previous studies on similar thin‐film WTaV demonstrated radiation resistance under ion irradiation [30]. The inclusion of V within the alloy was found to be the determining factor leading to radiation tolerance through influence of chemical short‐range order (CSRO) when compared to a W‐Ta ultrafine binary alloy (W_44_Ta_56_ in at%) [30]. However, the complexities and practical limitations associated with bulk synthesis often remain unaddressed in thin‐film studies [31, 32]. Thin films are typically few nanometer to micrometer thick and can exhibit confinement effects and enhanced grain boundary contributions, resulting in significantly different microstructure from bulk samples [33, 34]. Our current study demonstrates bulk synthesis of structurally and thermally stable WTaV alloy; a proposed advancement toward realizing W‐based compositionally complex concentrated alloys. Note that mechanical deformation mechanism at elevated temperature is beyond the scope of current study and we instead focus on bulk synthesis, phase stability, and thermal transport properties.

While W‐based RHEAs are recognized for their superior mechanical and irradiation properties relative to pure W [35, 36, 37], their inadequate thermal performance under fusion relevant service condition remains an obstacle to their applications as plasma facing components (PFC) [38]. Thermal conductivity is a crucial property of PFCs, and RHEAs typically exhibit thermal conductivity ranging from 1 to 45 Wm^−1 ^K^−1^ [39, 40, 41], while W exhibits a relatively higher thermal conductivity of 173 Wm^−1 ^K^−1^ at room temperature and 130 Wm^−1 ^K^−1^ at 1000 K [42]. However, while W‐based RHEAs retain thermal conductivity under irradiation, pure‐W is observed to lose significant thermal conductivity (up to 70%) at radiation doses of 0.6 dpa [38]. Therefore, a major target for the ongoing research is to address the trend of declining thermal conductivity with the increase of temperature for pure‐W and overall low thermal conductivity for RHEAs. Here, we show a major step forward in addressing this obstacle, where we not only show that WTaV retains a high thermal conductivity ∼57 Wm^−1 ^K^−1^ at room temperature, but also exhibits an increase in thermal conductivity with temperature, reaching as high as ∼134 Wm^−1 ^K^−1^ at 1000°C, rendering it the best thermal conductivity reported for RHEAs. Although pure tungsten exhibits higher thermal conductivity at room temperature, the WTaV alloy surpasses it at elevated temperatures, a critical advantage for high‐temperature applications where retention of thermal conductivity is paramount for optimum performance, thereby rendering WTaV more favorable than pure‐W for such environments. The uniqueness of superior thermal conductivity of the alloy has been scrutinized utilizing Density functional theory (DFT) calculations, which indicate that the presence of V in the WTaV alloy enhances transport properties, which lead to improved thermal performance, especially at higher temperatures. While the alloy is discussed in the context of extreme environments such as nuclear reactors, a detailed investigation of irradiation‐induced microstructural or thermal property evolution is beyond the scope of the present work.

## 2 Results

### 2.1 Alloy Design

A single‐phase body‐centered cubic (BCC) structure is preferred to avoid brittle intermetallic/secondary phases that would deteriorate the mechanical properties, especially at elevated temperatures [43]. As a first step to achieve this goal, thermophysical parameters were calculated for the composition of the alloy to determine if the composition is likely to retain a single‐phase BCC solid solution. Thermophysical parameter calculation involved the melting temperatures (T_m_), atomic size difference (δ), enthalpy of mixing (ΔH_mix_), entropy of mixing (ΔS_mix_), and Ω‐parameter (a function of T_m_, ΔH_mix_, and ΔS_mix;_ $\Omega =\frac{T_{m}\;.\;\Delta \mathrm{Smix}\;}{\left|\Delta \mathrm{Hmix}\right|}$) [44, 45, 46]. A single phase solid solution is reported to be achieved when the value of ΔH_mix_ is between −15 and 5 kJ/mol, Ω‐parameter >1.1, and δ is <6.6% [47, 48]. The formula and calculation details of these thermophysical parameters are discussed elsewhere [49, 50, 51, 52, 53]. The calculated thermophysical parameters for W_53_Ta_42_V_5_ are presented in Table 1. Additionally, valence electron concentration (VEC) was also calculated and tabulated [45, 54]. The value of VEC, when ≥8, implies that the material is likely going to maintain a FCC single phase structure while VEC ≤6.87 will lead to formation of a BCC solid solution [54].

**TABLE 1:** Thermophysical properties of W_53_Ta_42_V_5._

ρ g/cm^3^	δ %	ΔH_mix_ kJ/mol	VEC	ΔS_mix_ kJ/(mol.k)	T_m_ (K)	Ω	Crystal	Solid solution
17.48	2.52	−6.70	5.52	7.09	3445	3.65	BCC	Yes

**FIGURE 1:**
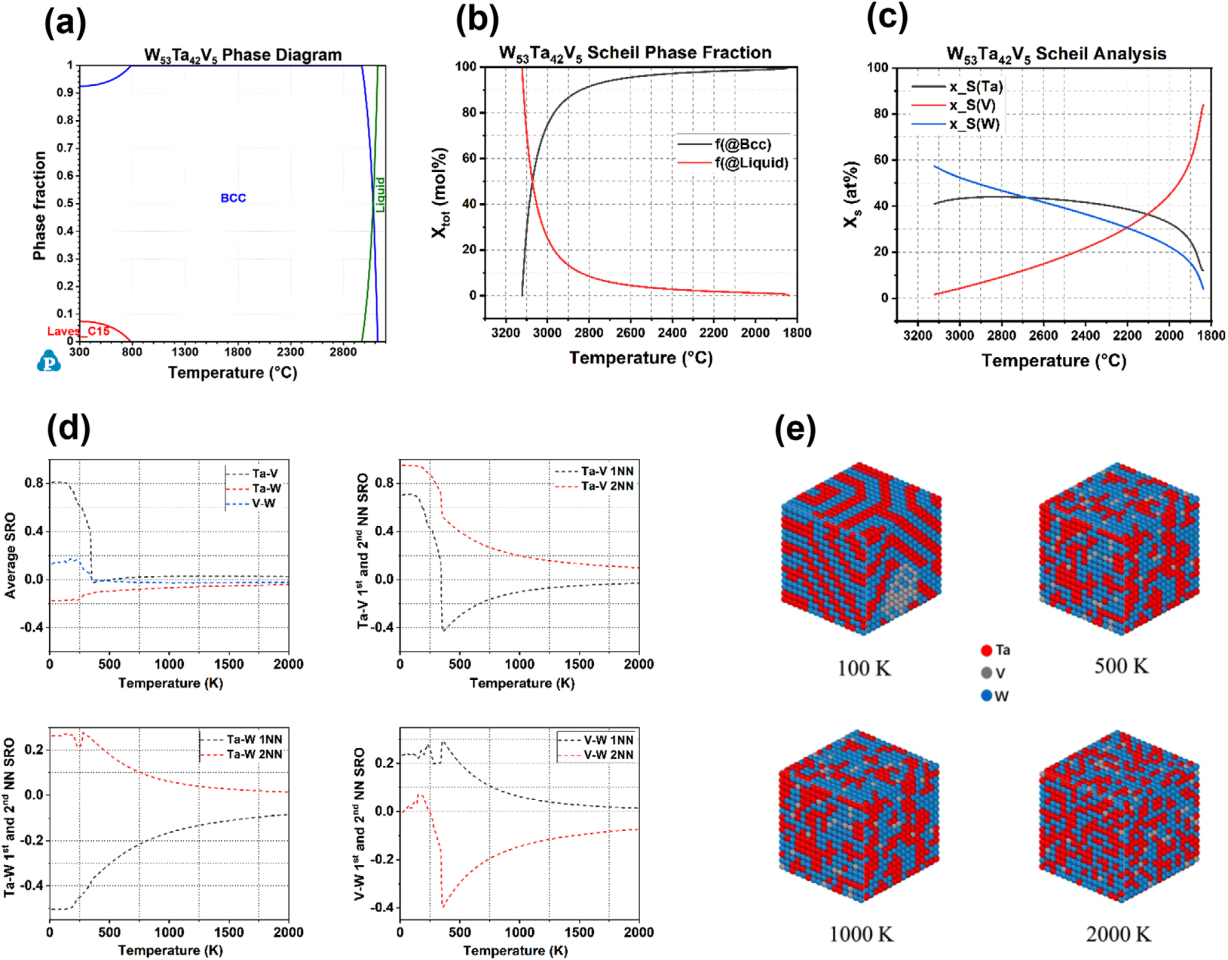
(a) Phase diagram of W_53_Ta_42_V_5_ alloy calculated using Pan_RHEA database in Pandat; (b) Scheil simulation showing phase fraction change with temperature during solidification; (c) Scheil simulation showing the fraction change of solid (W, Ta, and V) in liquid with temperature; (d) Atomic Monte Carlo simulation showing average chemical short‐range order and first and second nearest neighbor (NN) short‐range order for Ta‐V, Ta‐W, and V‐W in W_53_Ta_42_V_5_; (e) Atomic structures for W_53_Ta_42_V_5_ at 100, 500, 1000, and 2000 K.

The phase equilibria of the WTaV alloy was further scrutinized with the CALPHAD method [29]. The equilibrium phase diagram for W_53_Ta_42_V_5_ was determined using the PANDAT software with the Pan_RHEA database and shown in Figure 1a [55]. First, the equilibrium liquid range (the temperature between 100% and 0% liquid) is ∼150°C. It is also evident that a large BCC single phase region exists between ∼785°C and melting temperature, indicating thermal stability at higher temperatures. Below 785°C, part of the BCC phase decomposes into Laves_C15, the fraction of which increases with decreasing temperature with a maximum amount of ∼7.5 % at 300°C. However, the finding from CALPHAD is for the fully equilibrium state. The Scheil freezing curves (which is typical in arc‐cast samples) is shown in Figure 1b,c, exhibiting a much larger freezing range. However, no Laves phase formation is predicted, and the Ta content is expected to be relatively constant with segregation of V during the final stages of solidification as shown in Figure 1c. Additionally, from the equilibrium phase diagram from Figure 1a, the solidus temperature is T_s_ = 2971°C and the formation temperature of Laves_C15 phase is T_p_ = 785°C. The value of (T_s_—T_p_)/ T_s_ is 0.67, which is higher than the empirically predicted threshold value of 0.3 needed to facilitate the decomposition of primary phase [45, 52, 56]. As a result, decomposition of BCC phase is suppressed during cooling after homogenization at 1800°C, and a fully BCC solid solution is expected to be found in the material.

In addition to thermophysical parameter calculation and CALPHAD simulations, atomistic Monte Carlo (AMC) modelling was also utilized for alloy design. By leveraging a recently developed density functional theory (DFT) database for WTaCrVHf multi‐component system and a cluster expansion (CE) Hamiltonian, AMC simulations were performed to obtain information about thermodynamic properties and atomic structures [57, 58, 59, 60]. Figure 1d shows the average chemical short‐range order (CSRO) and first and second nearest neighbor (NN) short‐range order for binaries and atomic structures of the system at various temperatures. For the W and Ta system, the average of the first nearest‐neighbor (1NN) and second nearest‐neighbor (2NN) is negative. Therefore, it is expected that there will be dominance of bonding between W‐Ta system resulting from the negative values between 1NN and 2NN, which can also be complemented by the negative enthalpy of mixing for the binary of W and Ta [61]. For the W‐V pair, CSRO was found to be positive for 1NN and mostly negative for 2NN for temperatures above ∼350 K. Below 350 K, the CSRO becomes positive, meaning that at lower temperature, V atoms tend to stay away from W atoms. A similar trend is also observed for the Ta‐V system. Some clustering is expected because of Ta and V segregation since both elements belong to Group V and are expected to have positive enthalpy of mixing. Here, for the W_53_Ta_42_V_5_ alloy above 350 K, the average CSRO is expected to facilitate chemical stability through facilitating the random solid solution formation. Therefore, this AMC simulation predicts a more homogeneous atomic distribution and less probability of phase separation, especially at higher temperatures. However, due to positive average CSRO for Ta‐V at lower temperature, a tendency of segregation of V and Ta is likely to be observed, while the dominant phase will be the BCC matrix. Additionally, in Figure 1e, the AMC simulation shows the atomic configuration of 8192 atoms at 100, 500, 1000, and 2000 K. At low temperature (100 K), there is a tendency for V atoms to cluster together, but as temperature increases, the alloy tends to homogenize. Based on the findings from AMC simulations, as‐cast WTaV shows minor V segregation due to its lower melting point compared to W and Ta, while negative enthalpy of mixing between W and Ta promotes relatively uniform mixing in the predominantly disordered BCC solid solution. The addition of V suppresses potential WTa ordering, stabilizing solid solution, and high temperature annealing at 1800°C for 24 h eliminates V segregation.

Note that, The SRO analysis in the simulation is not meant to directly predict experimental SRO. In real materials there are many factors including impurities, defects, and processing methods that can introduce deviation between theoretical predictions and experimental observations. Nevertheless, these simulations are helpful for interpreting behavior that is observed experimentally and provide us insight into underlying trends observed by the experiment.

### 2.2 Synthesis

**FIGURE 2:**
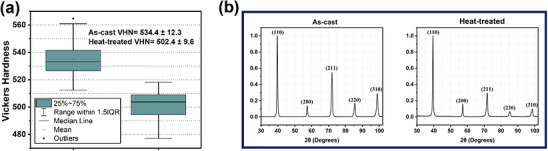
(a) Microhardness distribution of as‐cast and heat‐treated samples; (b) XRD results showing BCC single phase for both as‐cast and heat‐treated samples.

Utilizing arc‐casting, a fully dense WTaV sample was produced which was subsequently heat‐treated at 1800°C for 24 h. The manufactured sample is presented in an image in Figure S2. Because this alloy only used simplified composition based on three elements, materials and processing cost was reduced significantly compared to the other similar RHEAs containing four to six elements. The alloy was manufactured with standard arc‐melter and followed by standard heat‐treatment at 1800°C, which eliminated the need for advanced powder‐based processing or thermomechanical treatment. This straightforward process made the alloy more compatible with currently existing arc‐melting infrastructure and enhanced its potential for cost‐effective scale up. The sample was analyzed using XRD which indicated the presence of only a BCC phase within the microstructure, and Vickers hardness testing was performed for both the as‐cast and heat‐treated samples. The details of these findings are shown in Figure 2.

A hardness value of 534.4 ± 12.3 HV was measured for the as‐cast material. After heat‐treatment and homogenization, the hardness was measured to be 502.4 ± 9.6 HV, indicating a slight reduction of both the hardness value and their standard deviation (Figure 2a). This aligns well with previously reported hardness value for BCC/ B2 phase (between 500 and 600 HV) in similar high entropy alloys [62]. However, no secondary phase has contributed to establishing this elevated hardness, as only a BCC single phase is detected through XRD scans, which was further confirmed by the detailed transmission electron microscopy (TEM) analysis. Additionally, a grain size increase through heat‐treatment (discussed in the later part of the manuscript), has minimal impact on hardness value. Furthermore, as can be seen in Figure S3, indents on the grain boundary or very close to the grain boundary have negligible influence on hardness value. Therefore, without the major contribution from grain boundary strengthening (Hall‐Patch strengthening) and secondary phase strengthening, these results strongly suggest that the high hardness originates primarily from lattice distortion and the complex energy landscape characteristic of HEAs [14]. Importantly, this outcome demonstrates the successful synthesis of bulk WTaV alloy with good mechanical performance. The reduced standard deviation for hardness in the heat‐treated condition further indicates effective homogenization of the microstructure. Collectively, these results underscore the alloy's promise for high‐temperature structural applications.

In Figure 2b, in addition to showing only BCC crystal structure for the as‐cast and heat‐treated samples, the presence of minor texture was observed through XRD scans. While both as‐cast and heat‐treated samples display a dominant peak at (110) plane, the as‐cast sample showed higher intensity peaks at (211), (220), and (310) planes compared to the heat‐treated samples. This indicates a potential texture in some of the planes. Since the heat‐treated sample went through an annealing at 1800°C for 24 h, the grains were stress relieved and went through grain growth process, leading to altering the texture in the microstructure [63, 64, 65, 66]. The lattice parameter calculated from the XRD peaks was found to be 3.360 Å. Based on composition, Vegard`s law predicts the lattice parameter to be 3.216 Å [67]. The actual lattice parameter of alloys are often different than that predicted by Vegard`s law as it does not take lattice strains into consideration and only considers a linear rule of mixtures [68].

### 2.3 Microstructural Morphology

**FIGURE 3:**
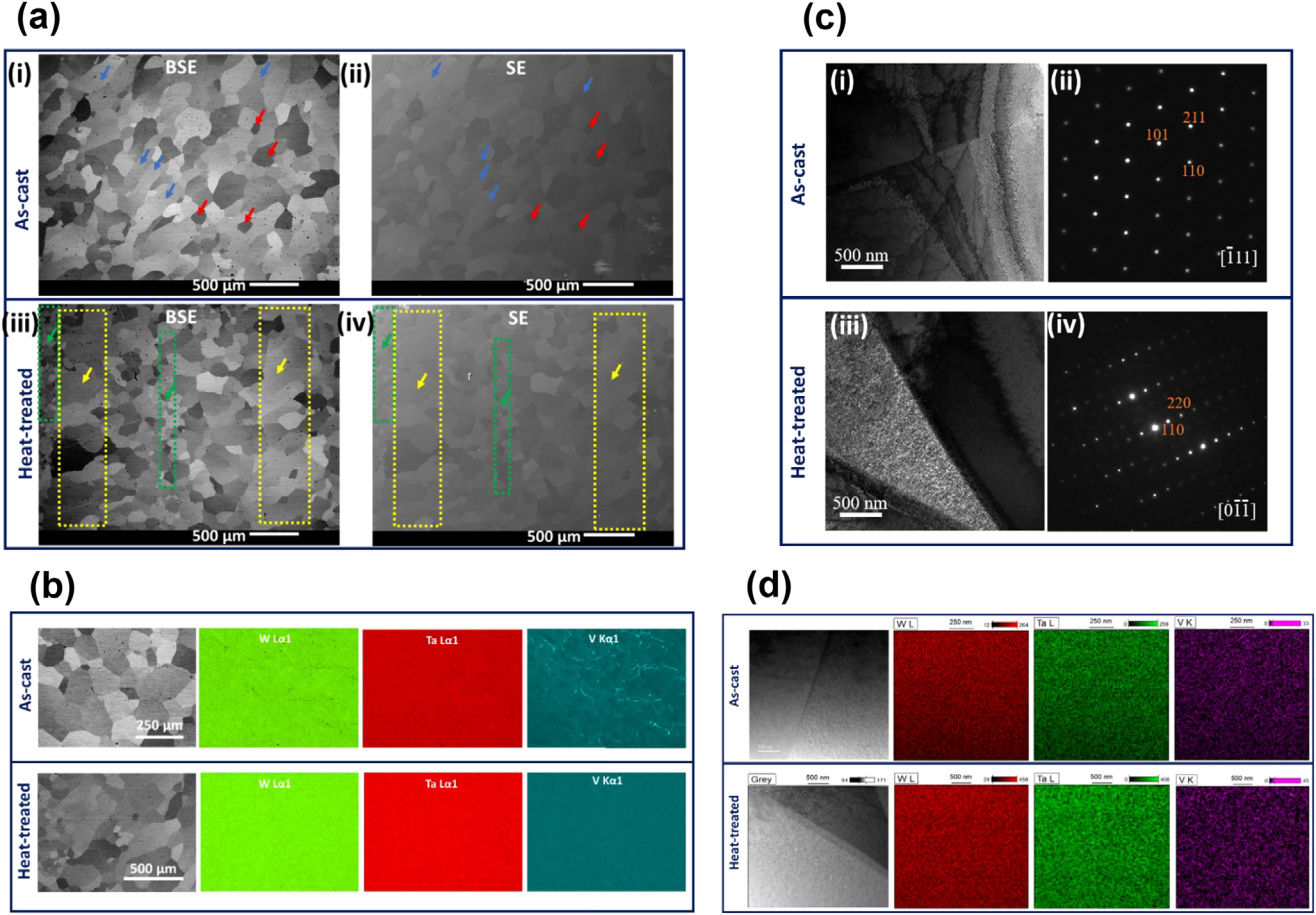
(a) BSE and SE images of as‐cast shown in (i) and (ii), respectively. BSE and SE images of heat‐treated samples shown in (iii) and (iv), respectively. In as‐cast sample, blue and red arrows showing elongated and equiaxed grains. (b) SEM‐EDS for as‐cast and heat‐treated samples showing elemental distribution; (c) TEM bright field image for (i) as‐cast and (ii) associated selected area diffraction pattern along ($\bar{1}\;1\;1$) zone axis, (iii) TEM bright field image for heat‐treated sample and (iv) associated selected area diffraction pattern along ($0\bar{\;1}$ $\bar{1}$) zone axis; (d) STEM‐EDS for as‐cast and heat‐treated sample showing elemental distribution.

Detailed microstructural analysis was conducted using scanning electron microscopy (SEM) and TEM, results of which are presented in Figure 3. Both secondary electron (SE) and back‐scattering electron (BSE) images for as‐cast and heat‐treated samples are presented in the Figure 3a. The grain morphology is clearly visible in both the BSE and SE images. For the as‐cast sample, both equiaxed (showed by red arrows) and elongated (showed by blue arrows) grains are visible. For the heat‐treated sample, different grain sizes are visible. Within the yellow box, the grains are much coarser while within the green box, the grains are of finer size. Further grain size analysis based on EBSD scan is discussed in the later part of the paper. Overall, for both the as‐cast and heat‐treated samples, a highly consolidated part was observed without the presence of any unmelted elements, and minimal solidification shrinkage was seen along the grain boundaries at the final stages of freezing cracks or porosity. Note that the dark spots visible in SE and BSE images in as‐cast and heat‐treated samples are polishing artifacts. Readers are referred to the Figure S1 in the supplementary file which confirms that these dark spots are residual Si resulting from polishing.

Elemental distribution through EDS of both the as‐cast and heat‐treated sample are shown in Figure 3b. As‐cast sample showed the depletion of W and enrichment of V in some of the grain boundaries, but not all. The distribution of Ta in the as‐cast material was uniform. The observed enrichment of V in some of the grain boundaries in as‐cast condition complements the AMC simulation presented in Figure 1e. This localized segregation of V along some of the grain boundaries possibly occurred during solidification, consistent with the Scheil analysis. However, no dendritic solidification was detected within the grains, possibly consistent with a low solidus/liquidus temperature separation. The heat‐treatment was successful in fully homogenizing the sample as no preferential segregation was observed in the heat‐treated sample. Average chemical composition was quantified based on the energy dispersive spectroscopy (EDS) spectrum for the whole map. The quantified compositional values are W 52.3 at%, Ta 42.7 at% and V 5 at%, which is close to the target composition of W_53_Ta_42_V_5_. It is evident that arc‐casting was successful in producing the bulk WTaV alloy.

TEM bright field images and the associated selected area diffraction (SAD) patterns for as‐cast and heat‐treated materials are shown in Figure 3c. Chemical distributions based on scanning transmission electron microscope (STEM)‐EDS for both as‐cast and heat‐treated samples, and results are shown in Figure 3d. The diffraction patterns correspond to BCC structure without the presence of any secondary phases, indicating that both as‐cast and heat‐treated samples retained a single phase and no intermetallic in the observed microstructure. While some V segregation was observed in SEM‐EDS analysis, for STEM‐EDS on both as‐cast and heat‐treated sample revealed no preferential segregation at nanoscale level. It is important to note that the segregation is only observed at some grain boundaries, not all. As a result, it is only visible when looking into a larger region in SEM, whereas focused ion beam (FIB) lift out for TEM was likely done from a grain boundary region which did not include such segregation, causing the discrepancies observed between SEM‐EDS and TEM‐EDS analysis. The uniform distribution in heat‐treated states for both SEM‐EDS and STEM‐EDS underscores the success of alloy design and processing methods in producing fully homogenized samples.

**FIGURE 4:**
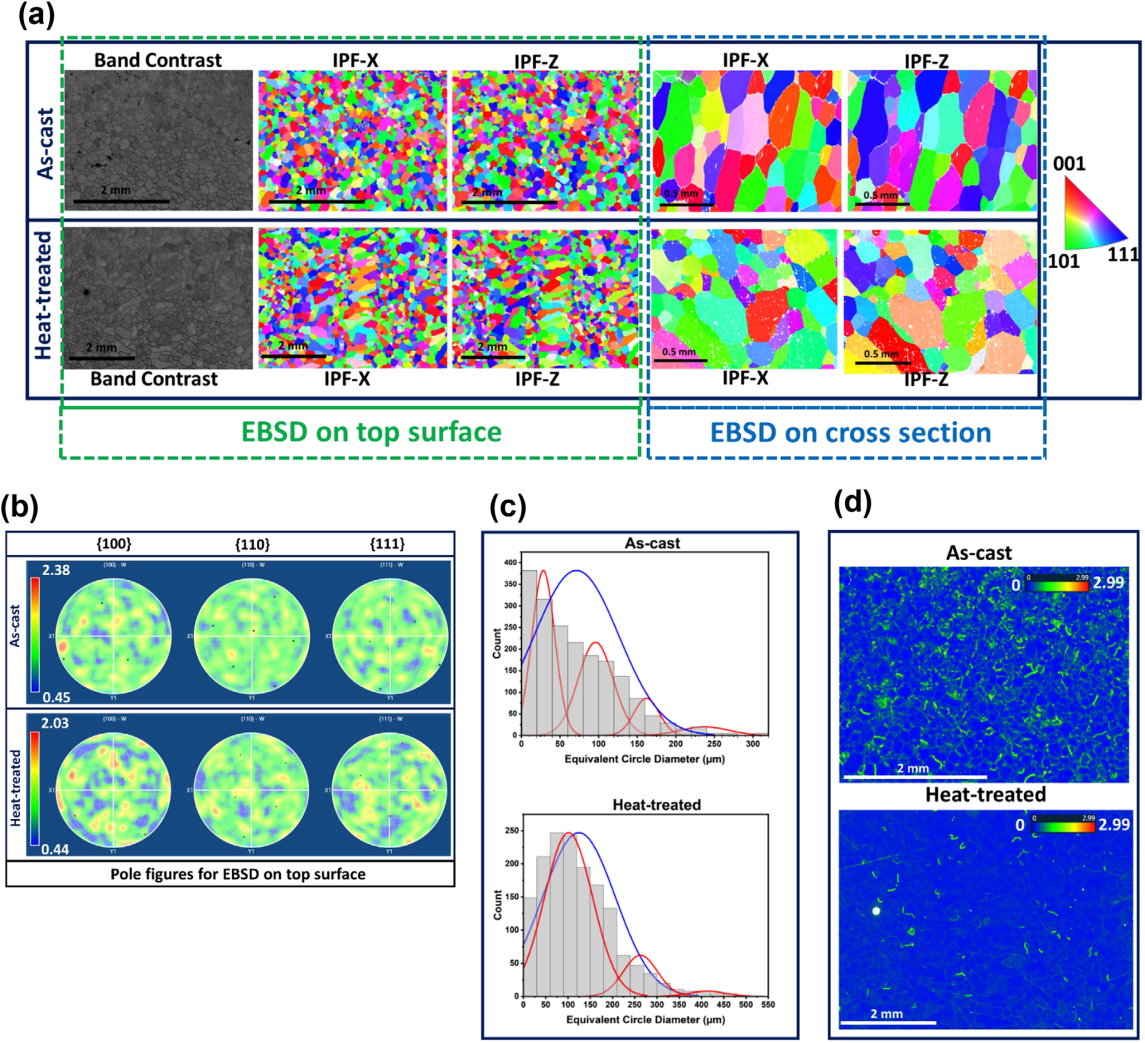
(a) For as‐cast and heat‐treated samples, EBSD results showing band contrast, inverse pole figure (IPF) along X and Z directions, EBSD scanning along both on top and cross‐section are included; (b) textures on top surface presented by pole figures along (100), (110), and (111) planes for as‐cast and heat‐treated samples; (c) Grain size distribution (for top surface) for as‐cast and heat‐treated samples based on EBSD scan where blue curve represents overall distribution of grain size and red curves represent each mode of grain size distribution; (d) KAM maps (for top surface) showing the difference in misorientation angle resulting from internal strains for as‐cast and heat‐treated samples.

Electron backscatter diffraction (EBSD) results including band contrast (BC) and inverse pole figures (IPF) for X and Z directions for both as‐cast and heat‐treated samples are shown in Figure 4a. EBSD scans on both the top surface and cross section are shown here. No major differences were evident from the EBSD scans along the two directions. Typically, during arc‐casting, solidification takes place along top to bottom surface and therefore, one would expect to see elongated grains along the cross section EBSD scan and relatively equiaxed grains along the top surface. However, no such difference in grain morphology is evident from the analyzed grains along the top surface and cross‐sectional surface. This indicates a relatively uniform solidification along all directions. Pole figures of both as‐cast and heat‐treated samples for top surface scan are shown in Figure 4b. The grain morphology is depicted by BC and IPF maps and the pole figures elucidate the crystallographic textures of the microstructure. The EBSD scans were indexed using a BCC‐W crystal data file. When matching with BCC‐W crystal file along the (100), (110), and (111) planes, the highest intensity was observed along the X1 direction (2.38 times random distribution), and minor contribution was noted in the Y1 direction (0.45 times random distribution). In comparison, for the heat‐treated sample, the intensity along X1 direction decreases slightly (2.03 times random distribution) while intensity along Y1 direction remains very similar (0.44 times random distribution).

Varying sizes of grains are visible in the BC and IPF maps (for top surface EBSD scan) in Figure 4a. Therefore, further analysis is performed here to calculate grain size distribution based on EBSD scans and is presented in Figure 4c. Note that, the arc‐cast samples were not uniform in size and therefore, different magnifications were used for as‐cast and heat‐treated samples during EBSD to capture the maximum region for grain size analysis. Here, evidence of multimodal grain size distribution is clearly observed. The blue colored curve shows the overall distribution of all the analyzed grains for both as‐cast and heat‐treated specimens. The red colored curves show individual distribution of each mode of grain size. To determine this grain size distribution, the procedure described by El‐Atwani et al. [69] was considered, and the analyzed results are shown in Table 2. The as‐cast sample has four modes of grain size distribution, the first mode that has a center at 28.38 µm and a total of 382 grains are located at this center. The centers of other peaks are located at 96.14, 162.17, and 237.63 µm. The last mode has the least number of large grains; with the highest grain count of 20 at the center of this distribution. A total of 1874 grains were analyzed for grain size distribution from EBSD scans on as‐cast sample. The heat‐treated sample had three modes with peak centers located at 101.34, 262.83, and 413.2 µm. The first mode had a total of 247 grains at its center and the last mode had a total of only 8 grains at its center. For the heat‐treated sample, the number of total analyzed grains was 1546. The largest grain size for as‐cast and heat‐treated sample are 317.36 and 523.27 µm, respectively.

**TABLE 2:** Values showing Gaussian fit of grain size distribution.

Sample	Peak	Location of center (µm)	Width (µm)	Height (Count)	Average grain size (µm)	Standard deviation (µm)	Total grains analyzed
**As‐cast**	1	28.38	29.54	382	**28.38**	14.77	1874
	2	96.14	45.24	216	**96.14**	22.62	
	3	162.17	32.78	86	**162.17**	16.39	
	4	237.63	61.98	20	**237.63**	30.99	
							
**Annealed**	1	101.34	108.02	247	**101.34**	54.01	1546
	2	262.83	76.48	62	**262.83**	38.24	
	3	413.2	78.52	8	**413.2**	39.26	

**FIGURE 5:**
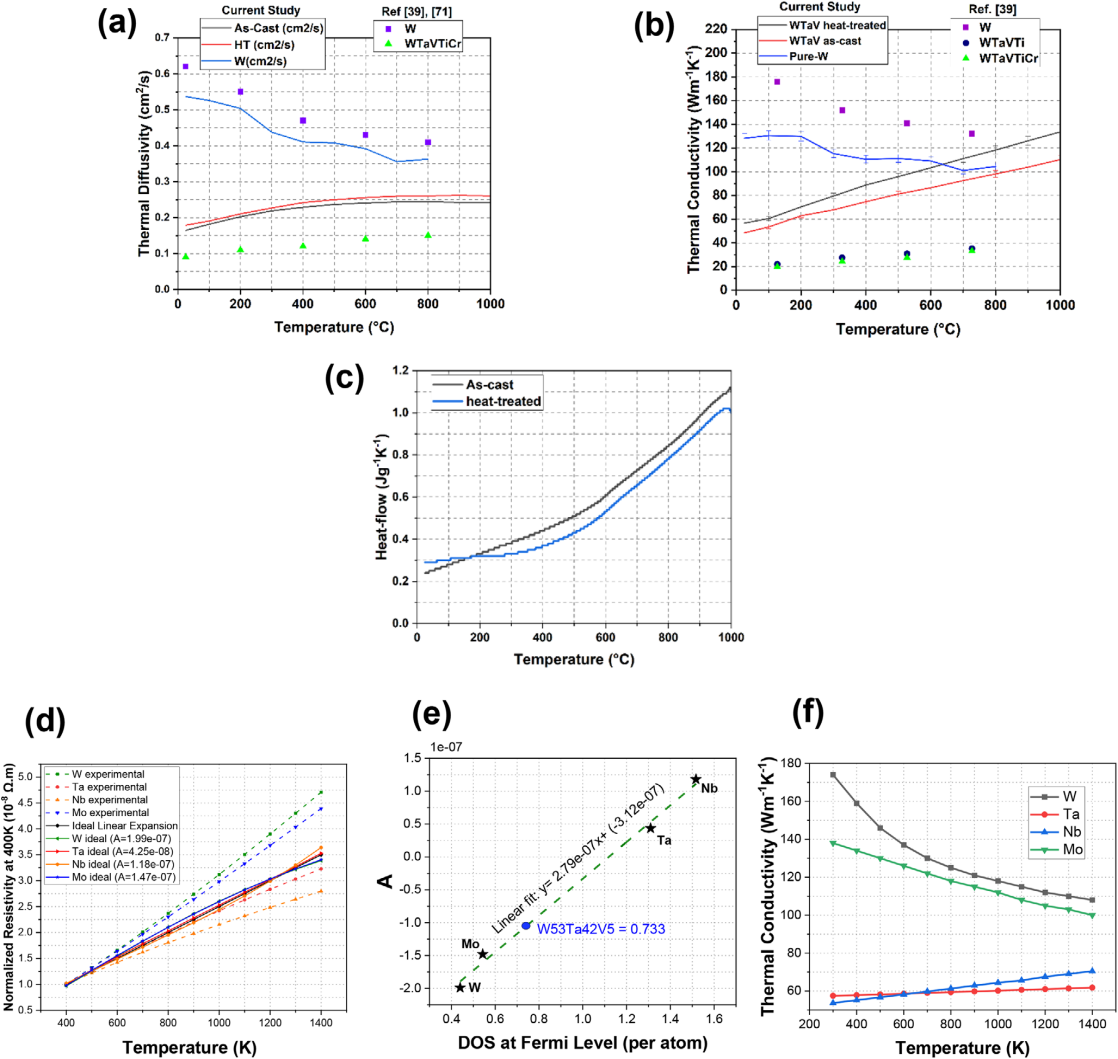
(a) Thermal diffusivity change with temperature for as‐cast and heat‐treated WTaV sample and pure‐W; (b) Thermal conductivity change with temperature for as‐cast and heat‐treated WTaV sample and pure‐W; (c) DSC curve showing change of absorbed/ released heat with temperature for as‐cast and heat‐treated samples; (d) Resistivity change with temperature normalized at 400 K for ideal and experimental values of W, Ta, Mo, and Nb; (e) Relation between value of *A* and density of states (DOS) at Fermi level per atom (f) Thermal conductivity change with temperature for W, Ta, Nb, and Mo.

To infer internal strain, the Kernel Average Misorientation (KAM) map was calculated for top surface EBSD scan and presented in Figure 4d for both as‐cast and heat‐treated samples. A 3^°^ maximum angle for KAM map and a Kernel size of 11 × 11 have been used for this analysis. It is evident that the as‐cast sample has higher KAM value, specifically along the grain boundaries, compared to the heat‐treated sample. Higher KAM value is associated with higher internal strain in the microstructure. This further suggests that the heat‐treatment at 1800°C for 24 h was successful in reducing residual stress originated during the solidification in arc‐casting process. Lower KAM values are also associated with recovery and recrystallization, where annihilated dislocations and strain free recrystallized grains contribute to a low KAM value. To further analyze phase transformations, differential scanning calorimetry (DSC) analysis was performed up to 1000°C for both as‐cast and heat‐treated samples and no evidence of phase transition was found, which is consistent with single phase BCC structure observed by XRD and microscopy. These DSC results are presented with other thermal analysis data in Figure 5C.

### 2.4 Thermal Analysis

Thermal diffusivities for as‐cast and heat‐treated WTaV samples are presented in Figure 5a and thermal conductivities for the as‐cast and heat‐treated samples are presented in Figure 5b. In both cases, experimentally measured thermal diffusivities and thermal conductivities of pure‐W are added here for comparison, showing the performance of WTaV samples compared to pure‐W. This also confirms the validity of the experiment as thermal conductivity of W is well known from literature [39]. Additionally, literature values for thermal conductivity and thermal diffusivity for pure‐W and similar HEAs are included in these figures. For both as‐cast and heat‐treated WTaV samples, a trend of increasing thermal conductivity is observed with rising temperature. This is likely resulting from the electron and phonon contributions at high temperatures [70]. At room temperature, as‐cast and heat‐treated samples had a thermal conductivity of 48.48 and 56.74 Wm^−1 ^K^−1^, respectively. These values increased to 110.30 and 133.63 Wm^−1 ^K^−1^, respectively, for the as‐cast and heat‐treated samples at 1000°C. The higher conductivity of the heat‑treated sample can be explained by the microstructural changes resulting from annealing at 1800°C. We observed from EBSD and EDS elemental mapping that heat‐treatment eliminated minor segregation which was present in the as‐cast state. KAM maps showed reduction of local misorientation and relief of internal stress. These changes reduced scattering of heat‑carrying electrons and phonons by compositional inhomogeneities, residual stresses, and lattice defects, leading to enhanced thermal transport in the heat‑treated sample. The grain size distributions of the as‑cast and heat‑treated samples are both heterogeneous, but heat treatment shifts the distribution toward larger grain sizes (Table 2 and Figure 4C). Grain boundaries act as scattering centers for phonons and electrons and thus, the coarser grain structure in the heat‑treated sample is expected to reduce grain‑boundary scattering and contribute to the observed increase in thermal conductivity.

Note that, the value of the conductivity (κ) is a function of the thermal diffusivity (D), specific heat capacity (Cp), and density (ρ) (κ=D×Cp×ρ). Diffusivity was directly calculated using the laser flash analyzer (LFA) method and the density was measured at room temperature using the Archimedes method, which was assumed to be constant for the investigated temperature range. Specific heat was determined up to 500°C and the rest of the Cp values up to 1000°C were determined from extrapolation using a second order polynomial equation. In all cases, the Cp values were found to be less than ∼25 Jmol^−1 ^K^−1^, which is the theoretical limit of specific heat of an alloy. In comparison, pure‐W had a thermal conductivity of ∼130 Wm^−1 ^K^−1^, but at 800°C the thermal conductivity was reduced to ∼105 Wm^−1 ^K^−1^. For pure‐W, specific heat capacity was directly found from differential scanning calorimetry (DSC) experiment. Literature values are also included in Figure 5a,b to show the superior thermal conductivity for WTaV alloy [39, 71].

When comparing to the literature values for pure W, reported thermal conductivity was found to be 173 Wm^−1 ^K^−1^ at room temperature, but the value decreased with temperature to ∼130 Wm^−1 ^K^−1^ at 1000 K [42, 71, 72]. The discrepancy between the conductivity of W measured here and the literature values is primarily due to specimen state (different heats with possible differences in texture, grain size and distribution, internal stress, etc.) and not the measurement error. Our W sample contained minor impurities and was not fully recrystallized. This can introduce internal stress and cause scattering of electrons and phonons, reducing thermal conductivity. In comparison, literature data are likely from high‐purity, fully annealed state under near ideal conditions. Factors like grain size, grain boundary density, and residual stress can significantly influence thermal conductivity. Nevertheless, the trend of decrease in conductivity with increasing temperature remains similar for both our experiments and literature reported values for W. On the other hand, W‐based multi‐component alloys are reported to have a wide range of thermal conductivity, though it is always lower compared to pure W. For example, at room temperature, WTaCrVTi high entropy alloy is reported to have a ∼20 Wm^−1 ^K^−1^ thermal conductivity [39] and TaTiNbZrX (X = Mo, W) has a thermal conductivity ranging from 5 to 45 Wm^−1 ^K^−1^ in the temperature spanning between 300 and 600 K [40]. On the lower end, as low as 1 Wm^−1 ^K^−1^ thermal conductivity was reported for (CoNi)_50_(TiZrHf)_50_ HEAs [41]. The reported reasons behind lower thermal conductivity of HEA include 1) additional scattering of electrons and phonons, 2) disruption of lattice periodicity from higher entropic configuration, and 3) presence of chemical short range order within the microstructure compared to pure alloys [70, 71, 73]. Compared to these RHEAs, the current WTaV has significantly higher thermal conductivity, especially at higher temperatures, making it good candidate material for fusion applications.

The DSC curves for both as‐cast and heat‐treated samples are presented in Figure 5c, where both samples are showing exothermic heat‐flow with rising temperature from room temperature to 1000°C. For the as‐cast sample, the increase of heat flow is gradual and smooth. No sharp endothermic and exothermic peaks were observed, which suggests the absence of distinct phase transformations within the investigated temperature range. The heat‐treated sample also showed a similar trend in DSC curve like the as‐cast sample, with gradual and smooth heat‐flow increase. However, the heat‐treated sample has slightly lower heat‐flow within most of the temperature range compared to as‐cast sample. The difference in relatively lower heat‐flow in the heat‐treated sample can arise from the changes in microstructure resulting from annealing. We learnt from the KAM maps (Figure 4d) that heat treatment caused a reduction in internal stress as well as influenced recovery and recrystallization within the microstructure. These microstructural changes influenced the ability of the alloy to absorb thermal energy, resulting in a lower heat flow.

## 3 Discussion

Refractory HEAs have potential use in applications within extreme environments due to their exceptional mechanical properties along with thermal and chemical stability. However, maintaining a single‐phase structure in the bulk form while retaining high thermal conductivity, remains unresolved due to issues such as intrinsic chemical disorder, potential for segregation, and difficulty of avoiding brittle intermetallic phases [74, 75, 76]. Here, we showed successful synthesis of a WTaV alloy in bulk form using arc‐casting, followed by heat‐treatment for microstructural homogeneity. The alloy not only retains a fully single‐phase BCC structure but also displays outstanding thermal and mechanical performance, setting a new benchmark for HEAs.

Manufacturing multicomponent concentrated alloys pose significant challenges due to differing thermophysical properties of the constituent elements and the inherent competition between entropy‐driven single‐phase stabilization and enthalpy‐driven phase separation [56, 77]. The successful production of WTaV alloy through arc‐casting is evident from the formation of full density, crack‐free ingots, where multiple remelting cycles during arc casting promote compatible melting and diffusion of the constituent elements, minimizing compositional segregation despite their differing melting temperatures. SEM, TEM, and EDS of the as‐cast samples revealed a primarily uniform microstructure, interrupted only by localized compositional inhomogeneities along grain boundaries. XRD further confirmed a single‐phase BCC structure, with no detectable peaks corresponding to secondary phases, ordered intermetallic, or phase‐separated regions. This result is particularly notable given the inherent complexity of multicomponent alloys. The formation of a stable single‐phase BCC structure suggests a favorable balance of atomic size differences, enthalpies of mixing, and overall configurational entropy—aligning with the thermodynamic criteria used to design our single‐phase alloy. Compared to prior literature, where arc‐cast HEAs often suffer from phase segregation or mixed FCC/BCC structures, our alloy demonstrates a significant leap in phase stability and structural control [11, 78].

From detailed microstructural characterization, a fascinating interplay of features is observed in the as‐cast condition which offers insights into the solidification characteristics. Most notably, multi‐modal grain size distribution was observed where grain size ranged from single digit micron to hundreds of microns. Local variation of the cooling rate or solute partitioning during solidification are likely reasons behind this observation [79, 80, 81, 82]. The surface of the arc‐cast sample is exposed to high cooling rate which promotes fine grains while slower cooling in the bulk region promotes coarse grains. The intermediate cooling rates in addition to grain boundary segregation and remelting during manufacturing result in additional grain populations with varying sizes. The coexistence of a range of grains from fine to coarse results in multi‐modal grain distribution. Heat‐treatment was successful to homogenize the sample with uniform elemental distribution. Nevertheless, the multi‐modal grain size distribution that was produced during the casting process, remains even after the heat‐treatment. Additionally, minor texture was observed, which may have originated by thermal gradients during arc‐casting [24, 83, 84]. A particularly interesting observation was the presence of minor elemental segregation within the as‐cast microstructure observed from SEM‐EDS, only occurring in a handful of grain boundaries. From a metallurgical point of view, in a typical scenario during arc‐casting solidification of W‐based high and medium entropy alloys, compositions with the higher melting point solidify first and arrange themselves in the dendritic regions, while inter‐dendritic regions are occupied by the lower melting point compositions [56, 77, 85, 86]. This behavior, however, is not extensive enough to produce secondary phases and may be indicative of sluggish diffusion kinetics. While in some cases this micro‐segregation may raise concerns for certain applications, here the segregation was localized and did not significantly alter the phase integrity or mechanical behavior. The uniform solidification with minimal dendritic micro‐segregation is most likely a result of a narrow equilibrium temperature difference between the liquidus and solidus, and also could be influential in avoiding solidification cracking in the high thermal gradients of arc‐casting.

After the homogenization heat‐treatment, a complete uniformity of the microstructure was observed. SEM and STEM EDS analysis confirmed the absence of any segregation or secondary phases. The previously observed texture in as‐cast sample was also reduced significantly. The grain size distribution, however, remained similar to as‐cast condition with multimodal distribution, albeit grain growth was observed. The misorientation angle within the grains was reduced significantly, indicating some recovery and recrystallization from the as‐cast microstructure. These results demonstrate that the alloy is thermally stable and responds well to post‐processing heat treatment which is a desirable feature for scaling to industrial applications where heat‐treatment is standard practice for tuning properties. This transformation from a slightly segregated microstructure to a fully homogenized, single‐phase BCC system is indicative of the robustness of this alloy system. It is also evident that any residual stresses or non‐equilibrium features introduced during rapid arc‐melting are fully alleviated through heat‐treatment. The fact that no new phases emerged during heat‐treatment further validates the thermodynamic stability of the single‐phase design.

A major aspect of our study is the high Vickers hardness in both as‐cast and heat‐treated conditions, suggesting strong mechanical properties. We observed ∼94% hardness retention even after 1800°C‐24 h heat‐treatment, which indicates excellent thermal stability. Addition of 5 at% V results in lattice distortion of 2.52% (δ value) compared to ∼2% in binary W‐Ta alloy, which can contribute to increasing the strengthening effect by impeding dislocation movement. On the other hand, due to V addition, weakening of the strengthening will occur as V is replacing Ta in the alloy and shear modulus of V is much lower than Ta. However, this trade‐off enables us to have a uniform microstructure after homogenization, promoting overall microstructural robustness. In the as‐cast state, the average hardness was ∼535 HV, which is comparable to hardness in other refractory multi‐component alloys [62]. For instance, the reported hardness is ∼450 HV for laser powder bed fusion WTa binary samples [87] and for arc‐cast technique, the hardness ranged from ∼380 HV to ∼615 HV for different amount of Ta addition to W [88]. The arc‐cast WTa binary showed higher hardness (614.4 HV and 582.6 HV for 40 at% and 50 wt% Ta, respectively) compared to our WTaV alloy. The likely reason for this difference is from variations in processing conditions, cooling rate, residual stress, and segregation in addition to compositional difference between the alloys. Nevertheless, the impressive hardness value in our WTaV alloy suggests that the underlying BCC matrix, despite minor elemental segregation and texture, possesses sufficient lattice distortion to inhibit dislocation motion. Interestingly, after homogenization, only a minor reduction of hardness was observed. Removal of segregation and texture typically leads to softening of materials, which was not observed in the annealed sample. Additionally, recovery (evident from grain growth (from grain size distribution in Figure 4c and EBSD KAM maps in Figure 4d) in the annealed sample was also observed, all of which should typically lead to a decrease in hardness. However, the reduction of hardness resulting from decreased effect of grain‐boundary strengthening (Hall‐Patch), and dislocation strengthening in the annealed sample is minimal (only ∼30 HV). This observed behavior indicates that the strengthening mechanisms in this alloy are intrinsic, presumably originating from severe lattice distortion and solid solution strengthening [89, 90]. Note that, AMC simulation suggested the existence of some SRO within microstructure at low temperatures, however, SRO was not quantified experimentally in the bulk sample, and as‐such, its contribution to strengthening, especially in the homogenized state remains undetermined. It is important to note that presence of SRO results in diffused intensity peaks between diffraction spots, which were not observed in our alloy. Retained hardness implies that the mechanical integrity is not reliant on non‐equilibrium features, but rather on the chemical architecture of the solid solution. These findings are encouraging for structural applications, particularly in environments where thermal cycling or irradiation might otherwise degrade microstructure‐sensitive materials.

The most remarkable finding of this current study is the thermal conductivity, setting a new standard for BCC concentrated systems. Concentrated alloys are notoriously known for their low thermal conductivity compared to pure elements [39, 40, 41]. The basis of multicomponent concentrated alloys is entropic stabilization through the combination of few major elements, which not only influences the transport properties within the microstructure but also disrupts the uniform energy landscape found in a single element [41]. The presence of multiple elements causes significant lattice distortion, and having mass fluctuations results in phonon scattering by different species [91]. The random energy landscape also lowers the mobility of electrons [92]. Compared to high entropy alloys, our WTaV alloy has reduced entropic effects, lower atomic mass fluctuations, and a more uniform energy landscape.

In comparison to pure W, the thermal conductivity of WTaV is lower (∼57 vs 173 Wm^−1 ^K^−1^) at room temperature [42]. However, at higher temperature, W suffers from conductivity loss (130 Wm^−1 ^K^−1^ at 1000 K) while WTaV approaches a higher value of ∼134 Wm^−1 ^K^−1^ at 1000°C [42]. Since containing Ta and V in our WTaV alloy results in superior thermal conductivity compared to other W containing HEAs, it is evident that the transport properties in the alloy are enhanced. One way to explain this material behavior is through understanding the changes in the electronic band structure on addition of Ta and V. This is based on the framework put forth by Mott and Jones that the conduction electrons in transition metals have wave functions derived from the s and p states and that the electrical resistivity is mainly controlled by the scattering of these electrons in the d states due to electron‐phonon scattering [93]. This theory is successful in describing the deviations of the electrical conductivities of pure BCC refractory metals from the values predicted by the Bloch‐Gruneisen relation that predicts a linear relation with temperature above the Debye temperature (∼300 – 400 K for the elements in this study) [94, 95]. The electrical resistivity was observed to increase faster than expected for group IV and VI elements [95] which was later validated experimentally by Chiu et al. for V‐Cr and Ta‐W binary alloys [96]. Figure 5d shows the normalized electrical resistivity at 400 K of these elements. The mathematical formalism for predicting the electrical resistivity is based on the probability of the conduction electrons to scatter to d bands and is given by [93, 96]:

(1) $$\rho =KT\;\left(1+6\;\alpha \gamma T\right)\left(1-AT^{2}\right)$$

where α is the coefficient of thermal expansion, *K* is an alloy specific constant derived through experimental means, and γ is the Gruneisen constant. *A* is a stochastic parameter that is related to the probability of electrons scattering into d orbitals. The theory proposed that due to the localized nature of d orbitals, they act as traps for electron mobility and hence result in increased resistivity. We base this work on the assumption that this parameter *A* is a material property and the fact that it is related to the electronic density of states (DOS) at the Fermi level [93, 96]. We compute the *A* values for pure elements by plugging the normalized experimental data into the functional form of equation (1) and plot them against the DOS at Fermi level to fit a linear curve with excellent correlation (R = 0.99 & p = 0.003) as shown in Figure 5e. DFT methods were used to compute the DOS for these elements at the Fermi energy. We use this fit to compute the *A* values for our alloy and due to the linearly increasing nature of the curve, we observe that materials with lower DOS at the Fermi level will have a lower value of *A* and hence a higher probability of these scattering events, resulting in an accelerated increase in resistivity with temperature. Using the linear fit and by computing the DOS at Fermi level, we obtain the *A* parameters for our concentrated alloy. It must be noted that the parameter *A* exclusively contains information about the trends in electrical resistivity with temperature relative to the ideal resistivity and does not tell us anything about the absolute values. In fact, one of the ways to interpret the mathematical form of *A* is that metals with maximum DOS at Fermi level (Nb and Ta) are likely to have a high value of resistivity at temperatures just over the Debye temperature than metals with a low DOS at Fermi level, but will see a relatively decelerated increase in resistivity with temperature [93, 96]. Another interesting trend observed in these transition metals is that the thermal conductivity of group VI elements (W and Mo) goes down with temperature while group V elements (Nb and Ta) see an increase with temperature [97] as shown in Figure 5f. The data of Figure 5f is derived from reference [95]. This trend can now be conveniently explained by coupling the previously mentioned trends in resistivity with the Wiedemann‐Franz relation [98, 99, 100]:

(2) $$\kappa_{e}=\frac{LT}{\rho }$$

where κ_*e*_ is the electronic thermal conductivity, *L* is the Lorenz number, and ρ is the electrical resistivity. The total thermal conductivity κ is given by [101]

(3) $$\kappa =\kappa_{e}+\kappa_{l}$$

where κ_*e*_ and κ_*l*_ are the electronic and thermal contributions. The Lorenz number L is a value that is roughly constant for metals and metallic alloys as observed experimentally in binary and ternary transition alloy systems at temperatures above their Debye temperature (∼300–400 K) [102, 103, 104], and that allows us the write equation (2) as:

(4) $$\kappa_{e}.\rho =LT=f\left(T\right)$$

This functional form translates to the conclusion that at any given temperature, the product of electronic thermal conductivity κ_*e*_ and electrical resistivity ρ is roughly constant for metal alloys at any given temperature above the Debye temperature. This is consistent with our computational results and also the experimental observation that alloys with lower electrical resistivity values at a given temperature have higher thermal conductivity and vice‐versa. We can therefore conclude that the change in electronic thermal conductivity with temperature depends upon how the resistivity changes. This observation is then coupled with the previously derived concept that allows us to predict the trends in resistivity with temperature using equation (1). Using this approach, we hypothesize that if the rate of increase in resistivity ρ is ‘slow enough’, the Widemann‐Franz law from equation (4) would predict that the electronic thermal conductivity κ_*e*_ would have to increase with temperature for the relation to hold. This also explains the increase in thermal conductivity with temperature as observed in metals like Nb, Ta and their alloys [103]. It is hence observed that in this work, due to the addition of V, the alloy shows a relatively high DOS at the Fermi level as seen in Figure 5e which results in a decelerated trend in resistivity vs temperature as compared to pure W, exhibiting behavior similar to group V elements, which causes the thermal conductivity to increase with temperature. A general observation for metals and alloys is that materials in which the thermal conductivity increases with temperature have low thermal conductivity and high resistivity at temperatures just above the Debye temperature (Nb and Ta), while alloys that show a decay in thermal conductivity with temperature start with high values of thermal conductivity and low resistivity at low temperatures (W and Mo) which further supports out hypothesis. A key detail, however, is that since this relation only accounts for the electrical resistivity and conductivity, this peculiar trend in thermal conductivity is assumed to arise primarily from κ_*e*_. Our DFT calculations show that the κ_*l* _ values for this alloy are relatively small as compared to κ_*e*_ and converge toward a very small value at elevated temperatures. These calculations are validated based on the observation that the computed specific heat, which is dominated by phonons at these temperatures, agrees with experimental measurements and follows the Dulong‐Petit law [105]. Also, since for metals at these temperatures, κ_*e*_ ≫  κ_*l* _, it is reasonable to assume that the electronic contribution largely governs the overall thermal conductivity, especially at higher temperatures [100, 101].

Another aspect that seems to be significantly contributing to this peculiar trend in thermal conductivity is the relatively low Ioffe‐Regel limit predicted in this alloy. As the temperature increases in a metallic material, the average mean‐free path of electrons becomes shorter. The Ioffe‐Regel limit is defined as the point at which the average mean free path of conduction electrons becomes comparable to the average interatomic distance of the crystal lattice. This leads to a thermo‐electric phenomenon known as resistivity saturation, where the resistivity of the alloy stagnates to a maximum value and does not change with increase in temperature any further. Our computational results show a similar behavior where the electrical resistivity of the alloy stagnates to about 20–23 µΩ·cm in the temperature range of 300—1300 K. This saturation behavior is commonly observed in transition metals experimentally [106] but what's interesting is that this alloy has a significantly lower Ioffe‐Regel limit than its constituent elements. We also find in our work that this alloy is expected to obey the Wiedemann‐Franz relation throughout this temperature range and this greatly enhances the κ_*e*_ with temperature. Together, the enhanced DOS at Fermi level along with the electrical resistivity saturation result in this rising trend of electronic thermal conductivity κ_*e*_ and correspondingly for the total thermal conductivity for this alloy. The details about the theoretical framework and DFT calculations have not been included in this work for the sake of succinctness and to not digress. The details can be found in a complementary paper [107].

In this work, we compared the thermal conductivity of WTaV against high entropy refractory alloys and W, because these alloys represent a major class of candidate materials for nuclear environment. However, it is important to emphasize that WTaV itself is a three‐component alloy. This work is significant for the fact that with the introduction of relatively simple composition enables the alloy to achieve superior high temperature thermal properties and mechanical performance, compared to both pure W, and W‐Ta based alloys, without relying on the effect of configurational entropy.

## 4 Conclusion

Here, we successfully demonstrated the bulk manufacturing of a WTaV multi‐component alloy through arc‐casting which achieved single phase BCC structure without any secondary phases. This bulk synthesis is a significant advancement over the previous work on similar composition thin‐films, often lacking scalability or bulk thermal stability. Microstructural characterization revealed the absence of secondary phases in both as‐cast and annealed conditions, confirming excellent thermal stability at elevated temperatures. The high hardness observed in the as‐cast state, coupled with minimal reduction after annealing, further validates the structural robustness and successful synthesis of the alloy. Most impressively, our WTaV alloy exhibited excellent thermal conductivity that shows a trend of increase with temperature increments, surpassing the thermal behavior of conventional refractory high entropy alloys and pure‐W at high temperatures. DFT calculations confirmed that the addition of V in the WTaV multi‐component alloy contributed to the transport property enhancement and eventually yield an exceptional thermal conductivity of the alloy.

This unique combination of scalability, structural simplicity, high temperature stability, and superior thermal performance makes WTaV multi‐component alloy a strong candidate for demanding applications. Currently, large scale manufacturing is underway and our future efforts will focus on evaluating radiation tolerance and mechanical behavior such as tensile, creep, and fatigue under different conditions to establish the technological readiness of this alloy.

## 5 Experimental Section

### 5.1 Fabrication of WTaV and Heat‐Treatment

W‐Ta‐V samples were produced via arc‐casting using high purity (99.95%) pieces of each element. High purity argon (99.99%) was used to backfill the chamber three times after evacuation to a pressure of 2.0 × 10^−3^ torr. The target composition was W53‐Ta42‐V5 (in at%). Localized segregation and inhomogeneity are common phenomena when W‐based HEAs are produced via arc‐casting [28]. To ensure complete mixing with no unmolten pieces, the samples were re‐melted four times and the manufactured sample was between 15 and 20 g. Additionally, one of the samples was wrapped in a tantalum foil and heat‐treated at 1800°C for 24 h under vacuum. The sample was furnace cooled.

### 5.2 Sample Preparation and Microstructure Analysis

Both as‐cast and heat‐treated samples were sectioned and mounted in epoxy, followed by grinding, and polishing for microscopy. JEOL IT‐500 SEM with dual Oxford Ultim Max EDS detectors were used to observe the morphology of surface and chemical composition of the samples. EBSD was carried out with JEOL IT‐800 SEM equipped with Oxford EBSD detector. During EBSD, a step size of 3 and 3.2 µm was used for the as‐cast and heat‐treated samples, respectively. EDS maps were acquired using 20 kV energy while maintaining a ∼50% deadtime.

TEM samples were prepared using ThermoFisher Quanta 3D‐field emission gun (FEG) Ga ion with a final thinning step using 5 kV. TEM and STEM investigations were conducted using JEOL GRANDARM TEM equipped with Centuiro EDS detector at 300 kV. SEM, EDS, and EBSD data were analyzed using AZtec and AZtec Crystal. TEM data was analyzed using DigitalMicrograph software and STEM‐EDS data was analyzed using Pathfinder software.

XRD experiment was conducted using Bruker D8 Discovery X‐ray diffractometer with Cu Kα radiation for both the as‐cast and heat‐treated samples. For the 2θ angles, for as‐cast and heat‐treated samples, increments of 17° and 20° were used, respectively. The dwell time used were 120 and 100 s, respectively, for the as‐cast and heat‐treated samples. For both the samples, rotation was maintained in the ϕ direction at 360°/min. Here, a voltage 50 keV and a current of 1000 µA were used. For both samples, the 2θ angles between 20° and 100° were analyzed and presented here.

### 5.3 Microhardness Testing

In addition to microstructure characterization, hardness values were determined using microhardness tester (Clark CM‐700AT Micro Hardness Tester) with a dwell time of 10 s and a load of 0.5 kg. FT‐ARS9000 HPPS‐ARS software package was used to analyze each indent to evaluate the hardness. A total of 25 randomly distributed indents were used to find an average hardness value for both the as‐cast and heat‐treated samples. A sample of five indents on heat‐treated sample is shown in supplementary file (Figure S3) to show the readers how the indentation places were chosen randomly across the sample that covered grain boundary and bulk matrix.

### 5.4 Thermal Property Analysis

Thermal diffusivity was collected using Linseis LFA1000/2800 Light Flash Analysis (LFA) System with Nd:YAF laser source and liquid nitrogen cooled InSb‐IR detector. Graphite furnace was used as a heat source and pyrometer was used for monitoring sample temperature. The pulse setting was 2 ms at 350 V and temperature range considered was from room temperature to 1000°C. The LFA system determines thermal diffusivity according to the ASTM E‐1461 standard and the measurement error is approximately ±3%. To compare the thermal conductivity values of WTaV, W with minor impurities was used under the same experimental conditions. Both as‐cast and heat‐treated samples were 6 mm × 6 mm shaped with a thickness of ∼2.75 mm. Before measurement the samples were spray‐coated on both sides with a thin graphite layer. The evaluation model (proposed by L. Dusza [108]) includes correlation for heat losses and finite pulse length effects.

DSC was measured using Linseis HDSC pt 1600 with a platinum DSC type S sensor under helium environment. A constant helium flow of 5 L/h was maintained during measurement. DSC measurement was carried out between room temperature to 1000°C. For both heating and cooling, constant rate of 10°C/min was maintained. The sample size for DSC measurement was 2 mm × 2 mm with a thickness of ∼0.9 mm and the weight was approximately 69.3 mg. After cutting the sample, no further surface preparation was done on the sample. Sapphire was used as the reference material, which had a diameter of 3.96 mm and thickness of 0.97 mm with a weight of 48.09 mg.

### 5.5 Modeling Technique

To validate the results computationally, we employed Monte Carlo simulations hybridized with a Cluster Expansion model of CrHfTaVW quinary HEAs [60] and set the fraction of Hf and Cr to 0. Each simulation began at 2000 K and cooled to 0 K in increments of 10 K. To have a good convergence, we applied 2000 equilibrium passes and 2500 Monte Carlo passes per atom for each temperature step and the energy tolerance was set to 0.0005 eV per step. Each simulation cell contained 16*16*16 BCC unit cells with 8192 atoms.

The Warren‐Cowley short‐range order (SRO) parameters were used to determine the order‐disorder transition of the system. SROs can be produced from the probabilities of pairs as:

(5) $$\alpha_{n}^{ij}=1-\frac{y_{n}^{ij}}{c_{i}c_{j}}$$

And the average SROs are:

(6) $$\alpha_{avg}=\frac{8\alpha_{1}+6\alpha_{2}}{14}$$

Where the α_1_ and α_2_ are first nearest neighbor and second nearest neighbor SROs.

### 5.6 Details of DFT Calculations

The ATOMSK [109] package was used to construct the bulk structures of the WTaV alloy and the ionic relaxation was done using the VASP [110] package at zero pressure with the box shape, size and volume all allowed to change with a 3 × 3 × 3 k‐mesh. The bulk structure unit cell contained 32 atoms and a band‐structure energy change cut‐off of 10e‐4 eV and an RMM‐DIIS optimization technique. This was then followed by a self‐consistent field calculation (SCF) with an energy cut‐off for plane‐wave basis of 500 eV and a sigma value of 0.1 eV using the Methfessel‐Paxton smearing using a 9 × 9 × 9 gamma centered k‐mesh. This was followed by a non self‐consistent field (NSCF) calculation using a 15 × 15 × 15 k‐mesh with the same energy cut‐off and a finer sigma of 0.05 eV using the tetrahedron Blöchl smearing starting from the charge density calculations from the SCF run. Finally, the VASPKIT [111] package was used to extract the DOS and Fermi energy from the output. All the DFT computations were carried out using GW_sv pseudopotentials based on projector‐augmented wave method (POTPAW_GW_sv).

## Conflicts of Interest

The authors declare no conflict of interest.

## Supporting information




**Supporting File**: advs75036‐sup‐0001‐SuppMat.docx.

## Data Availability

The data that support the findings of this study are available from the corresponding author upon reasonable request.
