# Supplementary material for: Unveiling a Bulk WTaV Multicomponent Alloy With Superior Thermal Properties and Manufacturability
Source: Adv Sci (Weinh). 2026 Apr 1;13(33):e22334. doi: 10.1002/advs.202522334 (PMC13271593; doi:10.1002/advs.202522334)
Supplement: Supplementary file 1 — Supporting File: advs75036‐sup‐0001‐SuppMat.docx. [file ADVS-13-e22334-s001.docx]

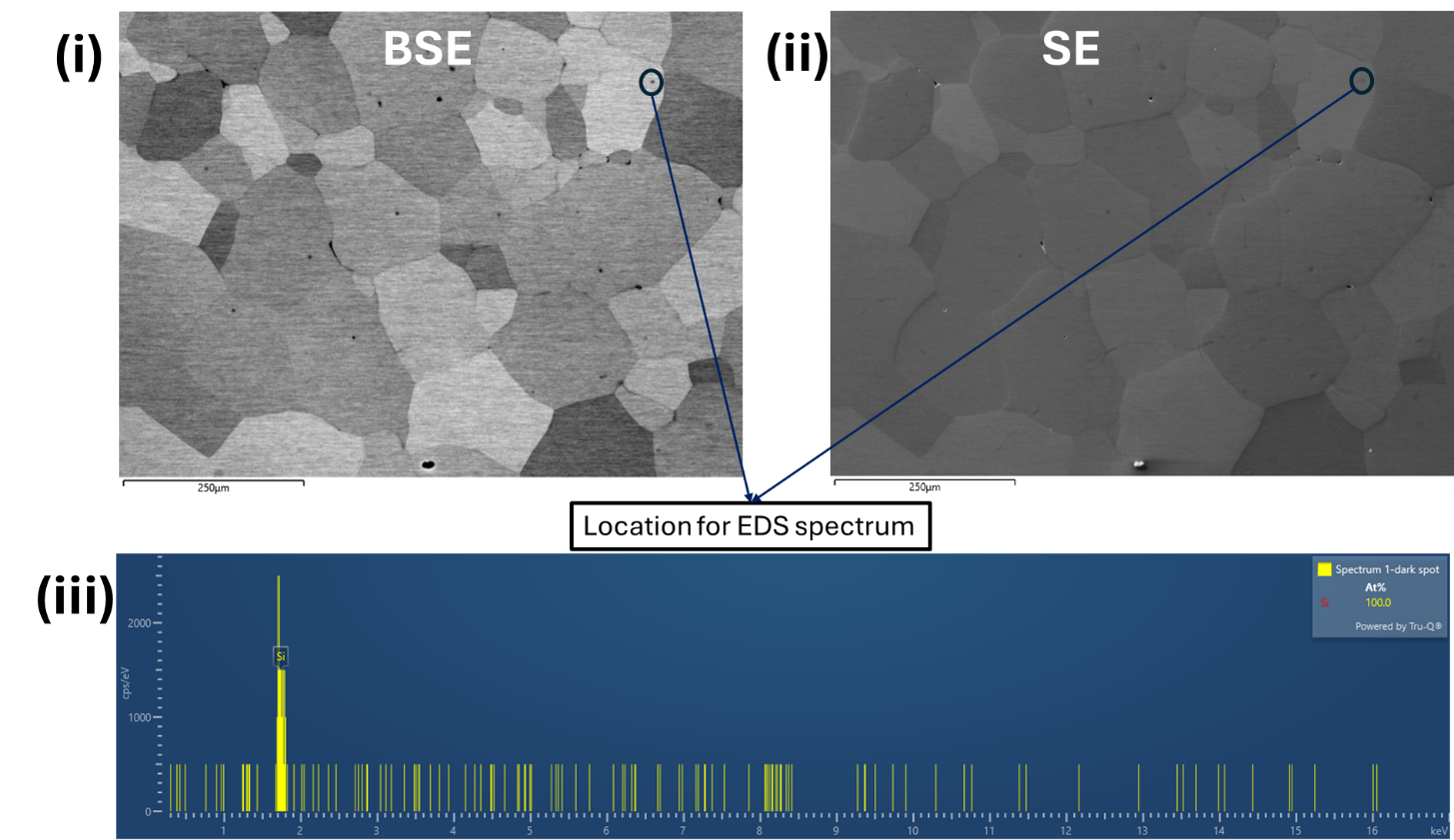
**Figure S1**

(a) BSE image and (b) SE image showing same location of as-cast WTaV sample. (c) showing EDS spectra from the dark spot within the marked circle indicating polishing artifacts

**Figure S2**


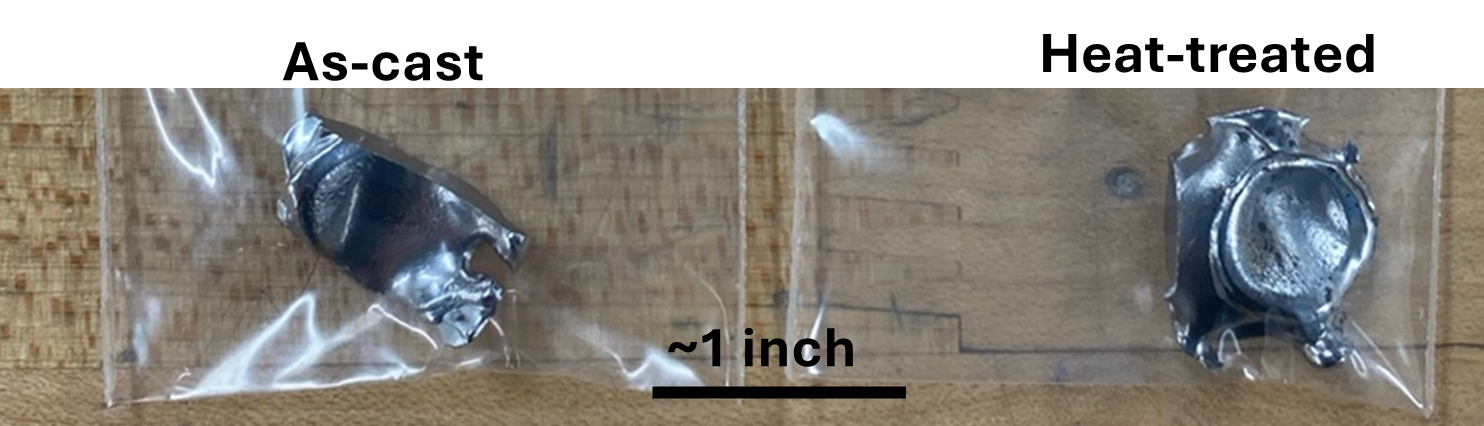


Images showing as-cast and heat-treated samples

**Figure S3**


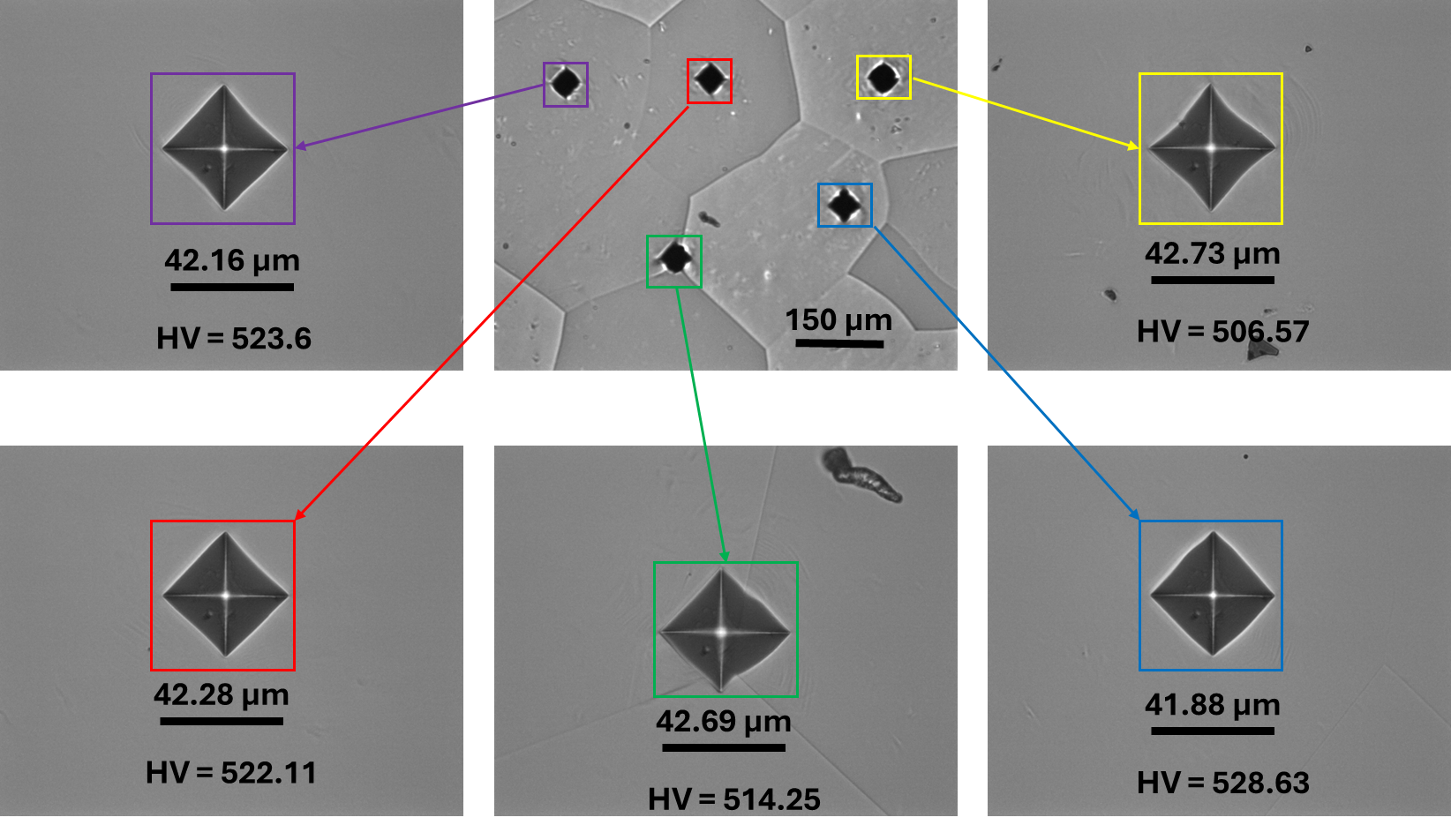


Five Indents on heat-treated sample are shown here to provide the readers an idea of how random indentations were used to acquire hardness data. Note that, this is not the actual hardness experiment presented in the main paper but a comparable hardness experiment.
